# Exploring the views of successful applicants for medical school about gender medicine using a gender-sensitive video assignment

**DOI:** 10.1186/s12909-020-1936-9

**Published:** 2020-01-28

**Authors:** Joni K. Scholte, Francisca W. M. van der Meulen, Theodora A. M. Teunissen, Mieke Albers, Roland F. J. M. Laan, Cornelia R. M. G. Fluit, Antoine L. M. Lagro-Janssen

**Affiliations:** 1 Department of Primary and Community Care, Gender & Women’s Health, Radboud University Medical Center, Post box 9101, 6500 HB Nijmegen, The Netherlands; 2 Radboudumc Health Academy, Research in Learning and Education, Radboud University Medical Center, Post box 9101, 6500 HB Nijmegen, The Netherlands

**Keywords:** Gender medicine, Sex, Gender, Undergraduate medical education, Curriculum development, Gender Bias, Sex/gender stereotyping, Reflection

## Abstract

**Background:**

Sex and gender influence health and disease outcomes, therefore, doctors should be able to deliver gender-sensitive care. To train gender-sensitive doctors, relevant sex and gender differences have to be included in medical education. In order to develop appealing, relevant, and effective education for undergraduate medical students, education should be tailored to students’ level and anticipated on their ideas and assumptions. Therefore, we wanted to answer the following research questions: 1. What do aspiring medical students want to learn about gender medicine?; 2. How would they like to learn about gender medicine?; and 3. What are their ideas and assumptions about sex and gender differences in health and disease?

**Methods:**

We performed an explorative thematic document analysis of educational assignments made by successful applicants (*n* = 50) during the selection procedure of their entry into medical school. To test aspirants’ capacity for self-directed learning, students were asked to formulate their own study plan after they watched a video that resembled a future practical experience (a consultation with a patient). As the content of this video was gender-sensitive, the assignments of the successful applicants gave us the unique opportunity to examine aspiring medical students’ views about gender medicine.

**Results:**

Aspiring medical students were eager to start their training to become gender-sensitive doctors. They believed in better care for all patients and thought doctors should obtain gender competences during their medical training. Students preferred to start with acquiring basic biomedical knowledge about differences between men and women and continue their training by developing gender-sensitive communication skills in (simulated) practical settings. Students differed in their interpretation of the gender-sensitive video, some generalized potential differences to all men and all women. Teachers were considered as important role models in learning about gender medicine.

**Conclusions:**

We advise medical schools to teach gender medicine from the beginning of medical school, by focusing on sex differences first and adding gender related themes later on in the curriculum. As students may interpret gender-sensitive information differently, structurally embedding reflection on gender medicine with gender competent teachers is necessary.

## Background

Sex and gender differences in health and disease are extensively discussed in recent literature [1–4]. Sex differences in the functioning of the reproductive systems are considered in health care, however, a wider range of biological factors and social, psychosocial, and cultural factors influence the health of men and women and also need to be taken into account [5–7]. Gender medicine strives to generate, apply, and implement knowledge on sex and gender differences in medicine that is beyond reproduction. Sex differences are defined as the *nature* that differentiates men from women and gender differences as the *nurture* by Oliffe & Greaves [8].

Gender-sensitive health care can be achieved if doctors apply their knowledge of gender medicine and understand the role of their own gender in their profession [1]. To provide the best care to both their male and female patients, doctors need to know what sex and gender differences they should take into consideration in their practice. Subsequently, they must be able to reflect on their attitudes or stereotyped beliefs and must achieve the competences to bring gender-sensitive medicine into practice. Combined with persistent support from gender medicine research, the need to develop gender-sensitive curricula has become more and more widely acknowledged [6, 9, 10].

Gender medicine is included as a milestone, i.e. competency-based development outcome, in both national [11] and international competence frameworks [12], highlighting medical students’ need to gain an understanding of sex and gender differences in health and illness. During their medical study, students need to become aware of their norms and values and when they start working in health care continue to take these into account [11]. However, sex and gender differences are currently neglected in medicine and insufficiently integrated into the international and national medical curricula [7, 9].

Verdonk et al. (2009) reported the objectives of successful implementation of sex and gender health issues into medical curricula. For example, medical education should focus on both biomedical and socio-cultural differences between men and women; students should receive education on sex and gender differences in several study years (min. 2 years); and health issues involving relevant sex and gender differences should be included, e.g. coronary heart disease, depression, pharmacotherapy, and sexual violence and abuse [13].

Several studies have expressed the importance of integrating sex and gender issues into medical education [6, 7, 10, 14–16] and have focused on incorporating gender medicine in educational training programs, learning objectives, and the hidden curriculum [9, 17–19]. In establishing a gender-sensitive medical curriculum, practical support, such as accessible educational materials, is an important facilitating factor for implementing gender into medical education [9, 14, 20, 21]. Various projects, therefore, have identified, developed, and disseminated modules and educational materials on gender medicine [9, 22]. However, gender medicine education is a relatively young scientific domain and to our knowledge no attention has yet been paid to the evaluation of these educational materials.

To make sure teachers give adequate and inspiring instructions, we need to understand how educational assignments for medical students can cultivate a more gender-sensitive view and increase knowledge of gender medicine. Students enter a learning environment with conceptions about how the world works, mostly without being aware of their own ideas [23]. Values and norms in society shape students’ preconceptions about gender [24]. Although, gender may have been discussed in their earlier learning environments, as far as we know students have generally not learned to reflect on their implicit ideas.

Education will be more effective if it matches students’ learning objectives and preferred learning activities. By studying the views of medical students, it is possible to cater to students’ level and anticipate on their ideas and assumptions. In this study, we focused on exploring the views of aspiring medical students about gender medicine. With these insights we aim to improve education materials about gender medicine by making it more appealing and relevant for students and include instructions to reflect on implicit ideas and assumptions. We were specifically interested in the opinions and ideas of medical students before they started medical school, since they were not influenced by the medical curriculum.

To cater to the students’ level at the start of medical school, it is important to know how students approach an educational assignment on a gender-sensitive theme: what questions come up, what would they like to learn, and how would they like to approach their learning objectives. We were also interested in the ideas and assumptions students have on sex and gender differences in general and specifically in medicine. We defined the following research questions: 1. What do aspiring medical students want to learn about gender medicine?; 2. How would they like to learn about gender medicine?; and 3. What are their ideas and assumptions about sex and gender differences in health and disease?

## Methods

### Study design

We performed an explorative thematic document analysis [25] of educational assignments made by successful applicants during the selection procedure of their entry into a Dutch medical school in 2015. This qualitative study design was used to explore the opinions and thoughts of future medical students.

### Setting and procedure

**Fig. 1:**
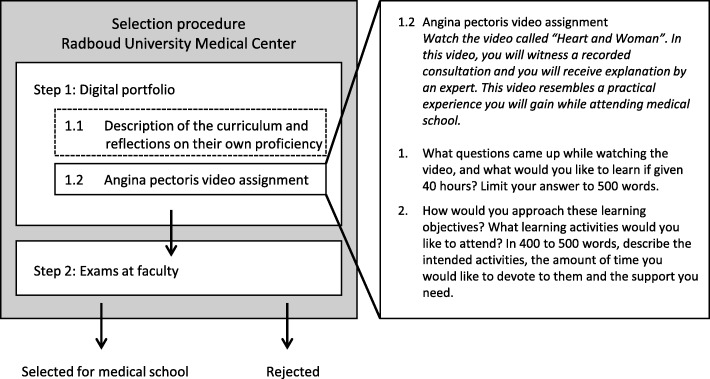
Selection procedure of medical school

At the Radboud university medical center (RUMC, the Netherlands), applicants for medical school were selected through a selection procedure in which applicants’ capacity to be successful in medical school was tested. In 2015, this procedure included two homework assignments (digital portfolio) and two exams at the faculty (Fig. 1). At the RUMC, students have to be capable to use their practical experiences to partly create their own study program (self-directed learning). To test their capacity for self-directed learning, aspirants were asked to watch a video that resembled a future practical experience (a consultation with a patient) and afterwards formulate their own study plan (Fig. 1, step 1.2 ‘Angina pectoris video assignment’). This plan included the formulation of learning objectives and learning activities they would like to attend if they were given 40 h (one study week). For both parts, applicants were limited in their word counts. The study plans of the applicants were merely assessed by the selection committee of the university on the basis of content and not on their attitudes/ideas about gender medicine. This content assessment included establishing students’ ability to formulate their questions, learning objectives, and learning activities.

The assignments of the successful applicants gave us the unique opportunity to examine medical students’ views about gender medicine before their enrolment in medical education. The gender-sensitive video was completely new and developed for implementation in the forthcoming years of undergraduate medical education, but had not yet been used. As said earlier, the video served as an example of a future practical experience for the portfolio assignment of the selection procedure and offered a perfect opportunity to study our research questions. Hence, the students watched the twelve-minute video called ‘Heart and Women’ (Additional file 1, Transcript video Heart and Women). This video showed fragments of a consultation of a general practitioner (GP) followed by a gender expert providing explanation regarding this consultation. The patient in the consultation is a 50-year-old woman with stress issues due to a combination of several stress factors: her husband may lose his job, she cares for her elderly mother, and she expects it may partly be due to menopause. Only when questioned by the GP does it turn out that the patient suffers from tiredness, vertigo, and shortness of breath on physical exertion such as cycling or vacuum-cleaning, these are pointed out as possible symptoms of angina pectoris in female patients. The gender expert explains gender differences in communication styles, in symptoms, and in the risk factors of angina pectoris.

### Participants

To be able to improve educational materials, we wanted to find out how and what future medical students wanted to learn about gender medicine. Therefore, for this study, we were merely interested in the views of successful applicants of the selection procedure, as these persons would enter medical school.

### Data collection

All applicants received an e-mail to evaluate the selection procedure of the RUMC. In this e-mail, applicants were asked for permission to use their assignments as research material for our study. Of the 621 applicants who submitted their portfolio assignment, 297 applicants were admitted to medical school via the selection procedure and were successful applicants. In total 76 successful students gave permission to use their assignments for our study. The response rate of successful applicants was 26% (76 of 297). At time of application, students were asked for their demographic characteristics (age and sex).

### Data analysis

We chose to analyse 50 of 76 assignments because we estimated that 20–25 documents (as commonly used in qualitative research) for qualitative analysis of documents with such a limited number of words would be insufficient to reach saturation. No variation other than age and sex was known and the age of the students was uniform. As we expected that male and female students would differ in their answers, we selected all assignments of the successful male applicants (*n* = 19) and randomly selected 31 of 57 assignments of the successful female applicants. We decided to select the assignments of the female students whose family names came first alphabetically.

The assignments were entered into Atlas.ti7, a software program for detailed coding in qualitative data analysis. We followed the analysing steps as described by Braun and Clarke [25]. This iterative process started with familiarizing with the data by reading the assignments and generating codes, then we searched for patterns by reading all the quotes that were coded, and reviewed these patterns in our data by reading the assignments again. We ended our process by defining themes. In our analysis, we paid specific attention to how gender was reflected in the learning objectives and activities that the aspiring students conceived.

The first phase of the analysis consisted of reading ten assignments. This was a random selection of the assignments of those students who had given permission for their assignments to be used as research material for our study. Key terms in the text were underlined and coded by inductive open coding by two researchers separately (FvdM and MA). Next, the researchers compared their code list and coded text segments. Differences in coding were discussed until consensus was reached. As a result, some codes were renamed, put together or split apart. No major disagreements emerged at this stage. A final code list was made by which all 50 assignments were coded line-by-line. Saturation was reached after analysing 30 assignments. However, to verify the saturation and warrant sex diversity, we decided to analyse the remaining chosen 20 assignments.

Next, two researchers (FvdM and CF) re-examined the codes and assignments to develop patterns deductively. These patterns were then discussed with all members of the research team (JS, FvdM, TT, MA, RL, CF, and ALJ) to construct themes. The quotations used in this study have been translated from Dutch to English by a bilingual speaker. In the results section, it is indicated whether the patterns had been brought up by a few (1–10), some (10–25), many (25–40), most (40–49), or all (50) applicants. In this study, we applied the consolidated criteria for reporting qualitative research (COREQ) [26]. Finally, demographic data (age and sex) were analysed using SPSS version 22.

## Results

### Descriptive statistics

We analysed the assignments of 50 successful applicants, consisting 31 women and 19 men. The mean age at the time of application was 18.4 years (female: 17–21 years; male 17–22 years).

### Overarching themes

Five overarching themes emerged from the students’ assignments. Two themes emerged from the first assignment concerning what questions came up and what aspiring students would like to learn: 1. Gender medicine as a self-evident part of doctor’s competence profile and 2. Eagerness to learn about gender. The next two themes emerged from the second assignment concerning how they would like to approach their learning objectives: 3. Basic biomedical knowledge first; and 4. The importance of teachers as role models. The last theme emerged from both assignments, concerning the ideas and assumptions students expressed in their assignments about sex and gender differences in health and disease: 5. Differences in interpreting gender medicine in health care.

### Gender medicine as a self-evident part of Doctor’s competence profile

All students felt that gender medicine was a self-evident topic that doctors should be able to handle in their future practice. In their eyes, a good doctor should be able to reassure both male and female patients and make them feel comfortable in the consulting room. Apparently, the students expressed no doubts that doctors therefore needed to have the appropriate theoretical knowledge as well as communication skills.

*I want to acquire appropriate communication skills to be able to help my patients properly in my subsequent career. In these lessons, I will focus on the correct way of addressing people and on learning to conduct an open conversation with both men and women. (P2, female)*

*It really matters a lot for a doctor to be aware of these [gender] differences. (P36, female)*

In their answers, all students focused on the patients’ gender rather than on their own gender, except for one student. She mentioned the influence her own gender might have on the doctor-patient relation.

*How can I reach out to patients of the opposite gender? […] I would like to be able to take the perspective of the opposite gender so that I can interpret their complaints properly. (P20, female)*

### Eagerness to learn about gender

After watching the video, most students stressed the importance of sex and gender in medicine and were instantly eager to learn more about this subject. A few students reported that they had never been aware of specific risk factors for women or men. A few female students also mentioned that they did not know about the existence of any gender differences in communication styles, which are relevant for medical practice. They declared that the video had raised several questions on sex and gender differences in other issues that possibly have to be taken into consideration in patient contacts.

*Are there any other diseases where women show different symptoms than men? (P20, female)*

*I am wondering what therapies there are, what medicines are appropriate and whether there are any differences between men and women in this area too. There might well be medicines that are not suitable for women when they are pregnant, for instance. (P8, female)*

The information presented in the video triggered different responses from the students. Some students asked questions about specific female risk factors, while few others, which were all female students, extrapolated the given case to the discussion of gender duality: differences may also influence treatment and health care outcomes for men.

*As there are so many differences between men and women, I am wondering in this specific case [angina pectoris] whether the incidence is higher in women than in men, or the other way around. I now know the main risk factors for women, but there will be some for men as well, I suppose. (P13, female)*

### Basic biomedical knowledge first

If they were given 40 h (one study week), most students preferred to gain basic biomedical knowledge of gender medicine concerning differences between men and women first and then acquire gender-sensitive communication skills. Many students mentioned that they want to obtain biomedical knowledge in an educational setting by reading background information from scientific literature and national guidelines, watching videos, attending lectures, writing research reports, or giving oral presentations. Few students mentioned that they actively want to learn by making a patient brochure, doing a research project, or discussing literature in group sessions.

*I will be doing a literature review with a couple of other students, and so I will be consulting medical sources and scientific papers. By talking about the subject, we improve our understanding of the content. And by allocating tasks, we can go more deeply into the content. Then we will produce a written report, an information leaflet, and a presentation on coronary atherosclerosis for women. (P29, female)*

With regard to psychosocial differences, many students focused to gain insight into communication styles and differences between men and women. They preferred to develop communication skills in practice, such as internships or simulated situations with other students or actors. In order to become gender-sensitive, it appeared essential to the students to be given feedback by peers, teachers, and patients.

### The importance of teachers as role models

All students mentioned teacher-centered education, such as attending lectures given by experts and asking teachers about their professional experiences. Doctors played a pivotal role as experts not only in teaching students through their observations and lectures, but also as coaches by providing feedback, answering questions, and guiding students’ learning processes. Overall, students considered teachers as role models.

*You will be watching an experienced doctor conducting a consultation with a patient. You should be paying attention to how the doctor communicates with the patient and compare this with what you have learned. Afterwards, you will assess and discuss the consultation with the doctor and you will be given the opportunity to ask questions. (P39, male)*

### Differences in interpreting gender medicine in health care

Students varied in their interpretation of gender. Some students assumed that the male body could be seen as normal and the female body as a deviation from this norm.

*In addition, I would look up how people communicate and in what way women deviate from this. (P37, female)*

Where the video said that women *might* present atypical symptoms and *might* present their complaints differently, some students generalized these differences to *all* men and *all* women. Besides, we found examples of stereotypical ideas in the students’ assignments.

*In this course, I would like to learn how I should communicate with patients: that I should spend more time talking to women, for instance. (P39, male)*

A few students indicated that they considered gender as being part of diversity. They also wanted to learn about different age groups, ethnic groups, patients with different cultures, personalities, and educational level, and were cautious in formulating specific aims to learn about differences between men and women.

*Next to that I need to know about the differences in symptoms, risk factors and their impact between men and women I also need to know about differences between age groups and ethnicities, so I will not be overlooking things when patients present their problems. (P49, male)*

A few students argued that a doctor’s first priority should be the best health care tailored to each individual patient. They observed that differences between patients should indeed be taken into account, but that specific attention to sex was unnecessary, as this patient-centered approach would automatically include differences between men and women. In other words, these students want to treat every patient as an individual taking irrespective of sex.

## Discussion

First of all, our study shows that students want to learn about sex and gender differences and that they are surprised about the role sex and gender play in health care. Moreover, we find that students identify themselves with their future role as doctors and think dealing with patients’ sex and gender differences is a self-evident part of their professional competence. Secondly, students prefer to learn about gender medicine by first gaining basic biomedical knowledge of gender medicine and then learn to communicate in a gender-sensitive manner by practicing with (simulation) patients. Furthermore, teachers have an important position in students’ learning activities. Thirdly, students showed different ideas and assumptions about sex and gender differences in health care including gender bias, generalization and stereotyping, considering gender as part of diversity, and treating every patient as an individual.

While our study shows that aspiring medical students respond with enthusiasm to gender issues in medical education, earlier studies revealed that there is considerable resistance to gender among health care professionals [7, 27, 28]. Celik et al. (2009) observed that professionals tend to be sceptical about the importance of gender in health care [27]. Nowadays, medical students grow up in a society in which gender equality is openly discussed [29], hence they might be more open to gender-related themes than older health care professionals. This would mean resistance may fade away over time. However, maybe students become less open to gender medicine at a higher level of medical school or when they start working. Therefore, the need to explicitly give and receive adequate feedback concerning gender issues from gender competent teachers and peers becomes even more crucial at the beginning of medical school. An international qualitative study by Mann et al. (2011) concluded that undergraduate students perceive feedback as an essential part of their learning process [30].

The answers of the students show that they first want to focus on basic biomedical knowledge about differences between men and women in an educational setting and then focus on acquiring gender-sensitive communication skills in a practical setting. This seems to be in conflict with modern medical curricula, in which real-life problems or tasks are the starting point, and learners are simultaneously integrating knowledge, skills, and attitudes. Maybe, beginning learners are afraid of excessive cognitive load when studying in a more integrated way [31].

In our previous study, medical teachers emphasize that the timing of introducing gender issues is crucial [14]. We believe that addressing gender at the start of medical school is effective in enhancing gender awareness.

In our study, students believe that teachers play a pivotal role in learning about gender medicine and see teachers as their role models. To provide adequate instruction and feedback, teachers should also be prepared to reflect on their own perspectives and assumptions on sex and gender. Earlier research argues that students not only learn from what is said, but also, when rethinking personal beliefs, take into account their teachers’ perspectives on gender [28, 32]. Therefore, teachers should be able to discuss students’ implicit attitudes and stereotyped beliefs [29, 33, 34].

Aspiring students show different interpretations of gender in the context of health care. They make connections with their assumptions about good health care, which is common for students at the novice level [35, 36]. Beginners are more likely than experts to approach problems by searching for correct explanations that fit their everyday intuitions [36]. They try to connect what they see with their prior knowledge and beliefs and values that are accepted in society. This results in a variety of interpretations of gender, including the idea that women are a deviation from the male norm, which is a form of gender bias [21], and the generalization of differences to all men and women (stereotyping). Therefore, it is essential that a gender assignment is openly discussed, to make sure students become aware of their own norms and values and take these into account when working in health care.

### Strengths and limitations

To our knowledge this is the first study to offer in-depth information on pupils’ knowledge of and views towards sex and gender issues before entering medical school. Examining these views from such an early stage provides interesting insights and helps to develop education that matches students’ own learning objectives and learning strategies in order to optimize learning about gender medicine. The strengths of our study is that we examined medical student’s views at a very early stage before entering medical school. As in the field of gender aspects stereotypes are very common, educational recommendations should not be based on the ideas derived from one perspective. To develop a gender-based curriculum the applicants’ suggestions should be combined with suggestions from experienced clinicians or experts in gender medicine. This study has some limitations. We explored answers to an assignment that was part of the selection procedure for medical school. This means that the aspiring students knew that their selection partly depended on their answers to this assignment, which may have provoked socially desirable answers about gender. Even though the gender-sensitive video assignment was only a small part of the selection procedure and the theme gender medicine was not specifically highlighted in the selection procedure, one cannot exclude social desirability bias, as applicants would not express a negative eagerness to learn about gender medicine. One also can argue that the title of the video “Heart and Women” indicates the gender aspect of the intervention which students directed (and biased) to emphasize on that. In this respect it would have been better if the video had received a neutral title. However, the video was intended to serve as an example of the knowledge that is required to reflect on this particular issue. The video provides factual knowledge and does not make any recommendations for the students’ learning objectives nor for any preferred learning strategies. In the assignment it was clearly stated that students had to make their own study plan based on the practical experiences they obtained by watching the video consult. We believe students focused on creating appropriate learning objectives and corresponding learning activities about gender medicine and felt that they could openly discuss their views and beliefs. Another limitation is that we could not use the assignments of the successful applicants who did not give us permission as input for our research. Next to this, as the students were limited in their answers by word count, moreover, they may have left out aspects, such as the consequences of gender inequality or their knowledge of other gender-sensitive health issues. Yet, the latter limitation could also be considered a strength, as students were forced to prioritize what they thought were the most important issues.

### Implications

By evaluating students’ questions, learning objectives, and learning activities about gender medicine, we gained knowledge of the ideas and views students have towards sex and gender upon entering medical school. Our study shows that students are eager to learn about sex and gender differences during medical school, but it also shows their different interpretations of gender including gender bias and stereotyping. In forthcoming research, it is interesting to compare the answers of the applicants with the experiences of senior clinicians or to contrast the students’ views with the results from a questionnaire to reveal existing gender stereotypes (triangulation). The Nijmegen Gender Awareness in Medicine Scale (N-GAMS) can serve as a validated questionnaire for this research. Furthermore, based on the results of our study, conducting a study with two different groups, one watching the video including the gender sensitive knowledge, and one group without, can be of great interest in order to assess the contribution of the gender sensitive video. Lastly, further research is needed to indicate if and, if so, how students’ ideas and assumptions about gender medicine change throughout medical school.

Teachers should be supported with educational materials that engage their students’ interests and that facilitate the process of their students becoming more gender-sensitive starting at the beginning of medical school. Moreover, medical teachers need to improve their gender teaching competences to be able to discuss sex and gender with students, to guide them towards gender sensitivity, and to redirect stereotyped perceptions. Teachers could be supported to improve their competences by organizing a ‘Teach the teacher’ gender training program on their medical faculty. In this training, teachers e.g. can follow an introductory e-learning and attend group meeting(s) which are guided by gender experts. This training program will help teachers to improve their knowledge, share their experiences with other teachers, and become aware of their attitudes regarding sex and gender. This awareness is important, as students not only learn about sex and gender in formal medical education. The so-called hidden curriculum is also an important determinant of students’ gender sensitivity [18, 32]. The training program should be explicitly targeted to both the female and male teachers of a medical faculty, as research of Risberg et al. (2003) showed that male teachers were more likely to avoid or simplify sex and gender issues and were less likely to have gender-sensitive attitudes [28]. Another possibility to support teachers is by endorsing existing gender networks [37, 38], which can facilitate collaboration between medical teachers on medical faculty level, on regional level, and national and international level. This collaboration can both be online via a website or applications or in real-life settings (e.g. conferences for medical education). Teachers can share educational materials and experiences.

## Conclusions

By offering aspiring medical students an educational tool, such as a gender-sensitive video assignment, they are encouraged to gain knowledge about sex and gender differences in health care. Most students feel that gender medicine matters and are interested to learn more about it. We advise medical schools to teach gender medicine from the beginning of medical school, by focusing on sex differences first and adding gender related themes later on in the curriculum. As students may interpret gender-sensitive information differently, structurally embedding reflection on gender medicine with gender competent teachers is necessary.

## Supplementary information

**Additional file 1.** Transcript video ‘Heart and Women’.

## Data Availability

The data used and/or analysed during the current study are available from the corresponding author on reasonable request.

## Abbreviations

COREQ: Consolidated criteria for Reporting Qualitative research
GP: General Practitioner
N-GAMS: Nijmegen Gender Awareness in Medicine Scale
RUMC: Radboud university medical center
