# Supplementary material for: Exploring the views of successful applicants for medical school about gender medicine using a gender-sensitive video assignment
Source: BMC Med Educ. 2020 Jan 28;20:25. doi: 10.1186/s12909-020-1936-9 (PMC6988225; doi:10.1186/s12909-020-1936-9)
Supplement: Supplementary file 1 — Additional file 1. Transcript video ‘Heart and Women’. [file 12909_2020_1936_MOESM1_ESM.docx]

**Transcript video ‘Heart and Women’**

| **Minutes of video:** | **Section of video:** | **Full transcript of spoken Dutch text or written Dutch text in video.** | **English sub titles of video. The text between brackets [..] is missing in the sub titles in the video.** |
| --- | --- | --- | --- |
| 00:00 – 00:03 | Text in video. | Cardiale klachten bij vrouwen  In gesprek met uw patiënt | [Cardiac complaints in women]  Talking with your patient |
| 00:07 – 00:24 | Voice over. | We weten inmiddels dat vrouwen met een hartaandoening andere symptomen kunnen ervaren dan mannen. | We now know that women’s symptoms **can differ** from those of men. |
|  |  | Bovendien presenteren vrouwen hun klachten vaak anders dan mannen. | Women [**often**] also present their complaints differently. |
|  |  | Omdat vrouwen hartklachten vaak pas ervaren in de leeftijd rondom de menopauze, worden de klachten makkelijk verward met overgangs- of spanningsklachten. | As women **usually** get cardiac complaints at menopause age they are often attributed to the change of life, stress. |
| 00:27 – 00:32 | Text in video.  *Mrs. Koning, the patient, walks towards the practice and takes a seat in the waiting room.* | Mevrouw Koning  - 50 jaar  - gehuwd, 2 kinderen  - administratief medewerker  - werkt parttime  - gebruikt geen medicatie  - geen chronische ziekten | Mrs. Koning  50, married, part-time job, no medication, no chronic ailments. |
| 00:32 – 00:37 | *General practitioner (GP) welcomes Mrs. Koning in the waiting room for her consultation.* | Huisarts: Mevrouw Koning, komt u maar. | GP: Mrs. Koning, please come in. |
|  |  | Mevrouw Koning: Dag dokter. | Mrs. Koning: [Hello doctor] |
|  |  | Huisarts: Goededag. | GP: [Good day] |
| 00:37 – 01:36 | *GP and Mrs. Koning are sitting in the consulting room.* | Huisarts: Wij hebben elkaar een tijd niet meer gezien. Wat kan ik voor u doen? | GP: It’s been a while. How can I help you? |
|  |  | Mevrouw Koning:  Nou, het gaat eigenlijk niet zo goed met mij. Ik ben nogal moe. Ik ben natuurlijk in de overgang en ik transpireer daardoor ook veel. | Mrs. Koning: I’m not feeling very well. I’m rather tired. My menopause has started, so I perspire a lot. |
|  |  | Huisarts: Ja. | GP: [Yes] |
|  |  | Mevrouw Koning: En hartkloppingen natuurlijk dat hoort er allemaal bij. Ja. Wat spanningen. Mijn man die denkt dat hij ontslagen gaat worden. Problemen op het werk. Reorganisatie. | Mrs. Koning: I get palpitations. That’s all part of it. And I’m under some strain. My husband thinks he’ll lose his job. Problems at work, a reorganization. |
|  |  | Huisarts: Ja. | GP: [Yes] |
|  |  | Mevrouw Koning: Mijn broer is een half jaar geleden overleden. Dus we hebben best wel een zware tijd gehad met elkaar. | Mrs. Koning: My brother died six months ago, so things have been tough for us. |
|  |  | Huisarts: Ja, dat snap ik. | GP: [Yes, I understand] |
|  |  | Mevrouw Koning: Door al die spanningen ben ik ook weer gaan roken. Want ja, het is gewoon veel. Ik heb ook de zorg voor mijn moeder nog. | Mrs. Koning: With all that stress, I started smoking again. It’s a big burden. I also take care of my mother. |
|  |  | Huisarts: Ja. | GP: [Yes] |
|  |  | Mevrouw Koning: Dus eigenlijk wil ik wel gewoon van die spanning af. Dat eh.. | So, I’d like to get rid of the stress. |
|  |  | Huisarts: Dat is uw vraag dan? | GP: [That’s your question?] |
|  |  | Mevrouw Koning: Ja. | Mrs. Koning: [Yes] |
|  |  | Huisarts: Uw broer overleden. | GP: Your brother died. |
|  |  | Mevrouw Koning: Ja. | Mrs. Koning: [Yes] |
|  |  | Huisarts: En uw moeder, zorgen voor uw moeder. Wat bedoelt u precies? | GP: And what’s the matter with your mother? |
|  |  | Mevrouw Koning: Nou ja, het gaat niet zo goed met haar, dus ik ben er vaak. | Mrs. Koning: She’s not well, so I often go there. |
|  |  | Huisarts: Ja. | GP: [Yes] |
|  |  | Mevrouw Koning: Ik help haar in de huishouding. Stofzuigen, schoonmaken. | Mrs. Koning: I help out with the housekeeping. Vacuuming, cleaning. |
|  |  | Huisarts: Ja. | GP: [Yes] |
|  |  | Mevrouw Koning: Nou, dat valt mij zwaar. | Mrs. Koning: It’s a big strain for me. |
| 01:36 – 01:46 | Text in video. | 1. Welke risicofactoren en symptomen heeft u gehoord? 2. Wat wilt u weten? 3. Hoe zou u nu verder gaan? 4. Wat zijn valkuilen voor de arts? | 1. Which risk factors and symptoms did you hear? 2. What do you want to know? 3. How would you continue? 4. What are the pitfalls for a doctor? |
| 01:47 – 02:15 | *GP and Mrs. Koning are sitting in the consulting room.* | Huisarts: Heel verhaal zo hé. Wat spanningen. Spanningen thuis, uw man, uw broer overleden en dan uw moeder, de zorgen om uw moeder. | GP: That’s quite a list. The stress. At home, with your husband. Your brother’s death, and the worries about your mother. |
|  |  | Mevrouw Koning: Ja. | Mrs. Koning: [Yes] |
|  |  | Huisarts: En u noemde ook heel wat lichamelijke klachten. Toch als ik uw verhaal zo hoor en u vraagt mij eigenlijk van, om er iets aan te doen. Wat kan er aan gedaan worden. | GP: And I heard you mention some physical complaints too. Your are basically asking me to do something about it. |
|  |  | Mevrouw Koning: Ja. | Mrs. Koning: [Yes] |
|  |  | Huisarts: Dan wil ik eigenlijk toch iets meer weten over de klachten die u heeft. En dat is met name om andere lichamelijke aandoeningen uit te kunnen sluiten. | GP: Tell me more about your complaints first. So I can eliminate other physical ailments. |
| 02:15 – 04:00 | *Gender expert: general practitioner and professor Gender & Women’s Health.* | Wat u net gezien heeft in het gesprek tussen mevrouw Koning en de huisarts, is dat de huisarts laat mevrouw Koning de eerste minuten gewoon praten. | What you’ve just seen in Mrs. Koning’s talk with her doctor, is that the doctor simply lets Mrs. Koning talk, for a few minutes. |
|  |  | Dat is natuurlijk voor iedere patiënt is dat goed, voor mannen en voor vrouwen. | This is good for any patient, both men and women. |
|  |  | Maar voor vrouwen is dat belangrijker nog omdat ze vaak zaken rondom hun eigen context vertellen, dus zaken die te maken hebben met hun relaties, en je ziet eigenlijk mevrouw Koning dat ook vrij snel doen. | But especially for women, as they often talk about matters of context. Concerning their relationships. You could see that Mrs. Koning did that fairly quickly. |
|  |  | Wat u ook heeft kunnen zien, is dat het een open communicatief gesprek was met veel open vragen, met veel meeleven, empathie, gevoelsreflecties heet dat. | You could also see it was an open, communicative conversation with a lot of open questions, a lot of concern and empathy. The emotions were being reflected. |
|  |  | Dus het was ook een gesprek waarin de doktor en de patiënt goed bij mekaar aan sloten. Wat ook heel belangrijk is dat de doktor aansloot bij de interpretatie van de klacht van mevrouw Koning. | So it was a talk in which doctor and patient connected well. What’s also very important is that the doctor responded to Mrs. Koning’s interpretation of the complaint. |
|  |  | Mevrouw Koning kwam binnen met het idee van ik heb spanningen en mijn overgang zal daar ook wel, overgangsklachten doen er ook niet goed aan. En ze wilde eigenlijk iets hebben voor die spanningen en daar sloot de doktor bij aan. Dus niet wat doktors vaker doen bij vrouwen, of het negeren wat de vrouw zegt of het ontkennen, tegenspreken. Dan voelt de patiënt zich helemaal niet serieus genomen. | She came in feeling that she was suffering from stress and that her menopausal complaints didn’t help. She wanted something to relieve the stress. The doctor responded to this.  He didn’t do what doctors often do with women: Ignoring or denying what they say. So they don’t feel taken seriously. |
|  |  | Dus hij ging daar wel op in maar volgde ook heel duidelijk zijn eigen pad en dat is wel iets wat in de communicatie met vrouwen een probleem kan zijn, echt een valkuil is dat vrouwen vaak al interpreteren dat iets door spanning of stress zal komen, en de doktor dat volgt, en dat doet deze doktor in ieder geval niet. | He responded, but he also followed his own strategy. This is something that can be a problem in communication with women. A pitfall. Women often assume the complaint is caused by stress and the doctor goes along with it. But this one didn’t. |
| 04:00 – 05:09 | *GP and Mrs. Koning are sitting in the consulting room.* | Huisarts: Dan wil ik eigenlijk toch wel iets meer weten over de klachten die u heeft. En dat is met name om andere lichamelijke aandoeningen uit te kunnen sluiten. En dan denk ik met name toch moeten uitsluiten dat het niet iets met het hart te maken heeft. | GP: I like to know a bit more about your complaints. Mainly because I want to eliminate other physical disorders. We should make sure first that your complaints aren’t related to your heart. |
|  |  | Mevrouw Koning: Oh! | Mrs. Koning: [Oh!] |
|  |  | Huisarts: Daar schrikt u van? | GP: This is a shock? |
|  |  | Mevrouw Koning: Ja. Daar heb ik zelf nog nooit aan gedacht. | Mrs. Koning: Yes. I’d never thought of that. |
|  |  | Huisarts: Nee. Vindt u het goed als ik u wat vragen ga stellen om toch wat meer inzicht te krijgen in de klachten die u heeft? | GP: [No] Can I ask you some questions, to find out more about your complaints? |
|  |  | Mevrouw Koning: Ja, prima. | Mrs. Koning: Yes, that’s fine. |
|  |  | Huisarts: Laten we beginnen bij het laatste stukje dat u vertelde. U moeder, het stofzuigen dat u daar doet. | GP: Let’s begin with the part about your mother. The vacuuming you do. |
|  |  | Mevrouw Koning: Ja. | Mrs. Koning: [Yes] |
|  |  | Huisarts: Dan beschrijft u dat u zich dan niet zo lekker voelt. | GP: You said you didn’t feel well doing it. |
|  |  | Mevrouw Koning: Ja, klopt. | Mrs. Koning: [Yes, that’s right] |
|  |  | Huisarts: Vertel daar toch is iets meer over. Wat merkt u precies? | GP: Tell me more about that. What do you feel? |
|  |  | Mevrouw Koning: Nou, een beetje draaierig, en gevoel dat ik een beetje benauwd ben. En dan ga ik even zitten en na een paar minuten dan gaat het wel weer wat beter. | Mrs. Koning: I get a little dizzy. Short of breath, I feel a little constricted. Then I sit down, and after a few minutes I feel a little better. |
|  |  | Huisarts: En andere momenten, zijn er ook andere momenten waarop u datzelfde ervaart? | GP: Are there any other times when you feel the same way? |
|  |  | Mevrouw Koning: Nou, ik had het laatst met fietsen ook. Toen moest ik afstappen en met de fiets aan de hand. Ja, want dat was eigenlijk hetzelfde gevoel inderdaad. | Mrs. Koning: The other day I had to get off my bike and continue on foot. Actually, it was the same feeling, yes. |
|  |  | Huisarts: Ja | GP: [ Yes] |
|  |  | Mevrouw Koning: Ja | Mrs. Koning: [Yes] |
| 05:09 – 06:36 | *Gender expert: general practitioner and professor Gender & Women’s Health.* | Een probleem bij het vertellen van klachten die te maken hebben met angina pectoris bij vrouwen is dat de klachten niet typisch zijn. | The problem with describing complaints related to angina pectoris, when it comes to women is that the symptoms are not typical. |
|  | Text in video between  05:20 – 05:25 | Typische beeld  1. Pijn op de borst uitstraling linkerarm | [Typical clinical picture  1. Chest pain with radiation to left arm] |
|  |  | U hoorde mevrouw Koning zeggen dat ze eigenlijk ook pas na goed doorvragen van de arts, van de huisarts, hoorde je zeggen dat ze draaierig werd. Ze voelde zich akelig, ze voelde zich naar, het gevoel kort van adem te zijn en moest dan gaan zitten. Met andere woorden de klachten die vrouwen presenteren, en die kunnen wijzen op angina pectoris zijn anders. | Only after the doctor kept asking more specific questions Mrs. Koning said that she sometimes felt dizzy. She got an unpleasant feeling, she felt bad. She also felt some shortness of breath. She had to sit down. So the complaints women describe, that could point to angina pectoris are different. |
|  | Text in video between  05:39 – 05:48 | Klachten bij vrouwen met coronair lijden:   1. Kortademigheid 2. Pijn tussen schouderbladen 3. Duizeligheid 4. Vermoeidheid bij inspanning 5. Diffuse klachten van pijn op de borst | [Complaints with women with coronary suffering:   1. Shortness of breath 2. Pain between the shoulder blades 3. Dizziness 4. Tiredness during physical exertion 5. Diffuse complaints of chest pain ] |
|  |  | Mevrouw Koning had wel, ook na uitvragen, eigenlijk na uitvragen, gaf ze duidelijk aan dat als ze moest fietsen zeker met wind tegen, dat ze dan wel dat akelige gevoel terugkreeg wat ze herkende van het stofzuigen. Dat is al redelijk duidelijk, het hoeft niet altijd zo duidelijk te zijn. En wat dan een hele goede tip is, is om vrouwen een dagboek te laten bijhouden, gewoon echt te laten opschrijven, wanneer ze dat nare gevoel krijgen, wat ze op dat moment aan het doen zijn, wat voor klachten ze daar nog meer bij hebben, of ze niet toch misschien druk op de borst  hebben en wat ze vervolgens doen en wanneer het dan weer weg is. Dus samengevat, twee dingen, het is vaak atypisch pijn op de borst of vaak is het zelf helemaal geen pijn op de borst. | Mrs. Koning did indicate after follow-up questions that she felt the same when riding her bike, especially against the wind. [That she then regained that unpleasant feeling] She recognized it form the vacuuming. That’s fairly clear already. It isn’t always that clear. A very good tip is asking women to keep a diary. To let them write down when they get that nasty feeling, what they were doing, and what other complaints they have. Is there tightness of the chest after all? What do they do, and how quickly do they feel better? So, two things: It’s **often** an atypical chest pain, or no chest pain at all. |
| 06:36 – 07:04 | *GP and Mrs. Koning are sitting in the consulting room.* | Huisarts: U zei net ook iets over uw broer. Uw broer is overleden nog niet zo lang geleden. | GP: You also mentioned your brother dying, not too long ago. |
|  |  | Mevrouw Koning: Ja. | Mrs. Koning: [Yes] |
|  |  | Huisarts: Wat was de reden dat hij overleed? | GP: What did he die of? |
|  |  | Mevrouw Koning: Een hartinfarct. | Mrs. Koning: A heart attack. |
|  |  | Huisarts: Een hartinfarct. | GP: [A heart attack] |
|  |  | Huisarts: En uw broer was volgens mij nog helemaal niet zo oud hé? | GP: And he wasn’t that old, was he? |
|  |  | Mevrouw Koning: 55. | Mrs. Koning: He was 55. |
|  |  | Huisarts: Dat is nog jong. | GP: Very young. |
|  |  | Mevrouw Koning: Ja, dat was zeker jong. | Mrs. Koning: It certainly is. |
|  |  | Huisarts: Is dat iets dat in de familie voorkomt? Hartproblemen? | GP: Does it run in the family? Heart trouble? |
|  |  | Mevrouw Koning: Nee, dat herken ik niet. | Mrs. Koning: I don’t think so. |
|  |  | Huisarts: Of hoge bloeddruk bijvoorbeeld? | GP: High blood pressure? |
|  |  | Mevrouw Koning: Ja, dat wel ja. | Mrs. Koning: There is that, yes. |
|  |  | Huisarts: Bij wie? | GP: Who had that? |
|  |  | Mevrouw Koning: Mijn vader. | Mrs. Koning: My father. |
|  |  | Huisarts: Ja. | GP: [Yes] |
| 07:04 – 08:16 | *Gender expert: general practitioner and professor Gender & Women’s Health.* | En dan is het dus heel erg belangrijk om een aantal risicofactoren goed te weten en ook goed de impact daarvan te weten. | It’s very important to be familiar with a number of risk factors and the impact they have. |
|  |  | Nou, bovenaan staat echt het roken. Roken is een risicofactor voor mannen én vrouwen, maar voor vrouwen in elk geval voor de overgang een grotere risicofactor. | Smoking is at the top of the list. It’s a risk factor for men and women. But a bigger one for women, especially before the menopause. |
|  |  | Het tweede is suikerziekte, diabetes mellitus, dat had mevrouw Koning niet maar diabetes mellitus is een risicofactor voor mannen én voor vrouwen op hartvaatziekten, maar voor vrouwen is dat risico 2 keer zo groot dan dat voor mannen is. | The second one is diabetes mellitus. Mrs. Koning didn’t have that. Diabetes mellitus is a cardiovascular risk factor in men and women. But the risk is twice as high for women as it is for men. |
|  |  | Dan het derde punt en dat is heel goed in dit consult uitgevraagd, dat is de familiare belasting. Geldt overigens voor mannen en voor vrouwen maar voor vrouwen wordt er vaak niet zo naar gevraagd omdat ze natuurlijk in de vrouwenlijn, oudere vrouwen veel minder rookten, dus je ziet dit snel als risicofactor bij mannen. Nou hier was een broer van 55 jaar overleden, en een vader met een hoge bloeddruk dus dat is echt een hele belangrijke factor. | The third factor, covered very well in this talk, is a hereditary defect. Both for men and women. But often women aren’t asked about it. Because in the female line, women used to smoke much less. So it came up more often with men. A brother who died at 55, and a father with high blood pressure are very important factors. |
|  |  | Dit waren dus de risicofactoren die voor mannen en voor vrouwen gelden, maar die in elk geval een andere impact hebben voor vrouwen. | These were risk factors that apply to both men and women, but have a different impact on women. |
|  | Text between  07:16 - 07:53. | Risicofactoren:  1. Roken  2. Diabetes mellitus  3. Familiaire belasting | [Risk factors:  1. Smoking  2. Diabetes mellitus  3. Hereditary defect ] |
| 08:16 – 09:00 |  | Daarnaast zijn er vrouwspecifieke risicofactoren. Nou, het belangrijkste is de pil. Ik denk dat weinig doktors zich realiseren dat de pil en met combinatie van roken, een risico is voor hartvaatziekten, maar oestrogenen natuurlijk ook. En dan zaken die heel erg gekoppeld zijn aan de zwangerschap, zoals een hoge bloeddruk in de zwangerschap, suikerziekte in de zwangerschap, en natuurlijk het HELLP-syndroom in de zwangerschap. En dan nog een hele andere die veel meer te maken heeft met het metabool syndroom wat bij vrouwen vaker kan voorkomen, dat is het polycysteus ovariumsyndroom, waarvan we nu denken dat het ook, de mate waarom weten we niet, dat dat ook een verhoogde risico geeft op hart- en vaatziekten. | There are also factors that are specific to women. Mainly, the birth control pill. Few doctors realize how big a factor this is, in combination with smoking. Estrogens as well, of course. Then there are factors that are closely linked to pregnancy. Such as high blood pressure and diabetes. And of course HELLP Syndrome. And another factor that has more to do with metabolic syndrome: Polycystic ovary syndrome. We think it also increases the risk of cardiovascular disease. But we don’t know by how much. |
|  | Text between  08:20 – 08:59 | Vrouwspecifieke risicofactoren:   1. Anticonceptiepil 2. Gebruik van oestrogenen 3. Zwangerschapscomplicaties 4. Polycysteus ovariumsyndroom | [Women specific risk factors:   1. Birth control pil 2. Usage of estrogens 3. Pregnancy complications 4. Polycystic ovary syndrome] |
| 09:00 – 10:13 | *GP and Mrs. Koning are sitting in the consulting room.* | Huisarts: U bent een paar weken geleden bij mij geweest. En naar aanleiding van de klachten die u toen had, heb ik u verwezen naar de cardioloog | GP: You came to see me a few weeks ago. Your complaints made me refer you to a cardiologist. |
|  | Text between  09:00 – 09:04. | 3 weken later… | [3 weeks later…] |
|  |  | Mevrouw Koning: Ja, dat klopt. | Mrs. Koning: [Yes, that’s right.] |
|  |  | Huisarts: U bent er inmiddels geweest, want ik heb een brief van de cardioloog ontvangen. U heeft een aantal testen gedaan, een fietstest, en hij heeft ook medicijnen voorgeschreven. | The cardiologist wrote to me, saying that the complaints are heart-related. You had some tests, and he prescribed medication. |
|  |  | Mevrouw Koning: Ja. | Mrs. Koning: [Yes] |
|  |  | Huisarts: Hoe gaat het nu met u? | GP: How do you feel now? |
|  |  | Mevrouw Koning: Beter. | Mrs. Koning: Better. |
|  |  | Huisarts: Beter? | GP: [Better?] |
|  |  | Mevrouw Koning: Ja, ik voel mij een stuk beter. | Mrs. Koning: I feel a lot better. |
|  |  | Huisarts: En de medicijnen die u heeft. Lukt dat om ze goed in te nemen? | GP: Can you manage to take the medication? |
|  |  | Mevrouw Koning: Ja, daar heb ik niet zo moeite mee. Dat gaat goed. | Mrs. Koning: Yes, that’s not a problem. |
|  |  | Huisarts: Dat is fijn. Wij hebben het de vorige keer ook over uw man gehad. U zei toen van, mijn man weet er eigenlijk niet zo van af. | GP: Good. Last time, we discussed your husband. You said he didn’t really know about this. |
|  |  | Mevrouw Koning: Nee. | Mrs. Koning: [No] |
|  |  | Huisarts: Hoe is dat nu? | GP: What’s the situation now? |
|  |  | Mevrouw Koning: Nou, ik heb op uw advies inderdaad met hem gesproken en daar ben ik eigenlijk wel heel blij om. Ik moet zeggen dat het mij ook wel heb opgelucht hoor. | Mrs. Koning: I took your advice and talked to him. And I’m very glad I did. I have to say it was a relief as well. |
|  |  | Huisarts: Ok. | GP: [Ok] |
|  |  | Mevrouw Koning: Hij schrok natuurlijk eerst wel, maar nu is het heel fijn en hebben wij het er over. | Mrs. Koning: It was a shock at first, of course. But now we discuss it, and that’s nice. |
|  |  | Huisarts: Fijn. | GP: [Good] |
|  |  | Mevrouw Koning: Ja. | Mrs. Koning: [Yes] |
|  |  | Huisarts: Ja, heel goed. Wat ik vandaag met u wil bespreken die we de vorige keer wel benoemd hebben, maar wij verder niet op in zijn gegaan. Die hebben te maken met leefstijl. U vertelde de vorige keer dat u weer was begonnen met roken. Daar wil ik het graag met u over hebben. Bewegen. | GP: Very good. Now I’d like to discuss some things we mentioned, but didn’t elaborate on. They have to do with lifestyle. You said you’d started smoking again. I’d like to discuss that. And exercise. |
| 10:13 – 10:57 | *Gender expert: general practitioner and professor Gender & Women’s Health.* | Wat wil ik dat u graag mee naar huis neemt als belangrijkste boodschappen, op de eerste plaats, bent u zich ervan bewust dat hartklachten ook bij vrouwen voorkomen. Op de tweede plaats, dat de presentatie van angina pectoris en van een infarct bij vrouwen anders is dan bij mannen, anders kan zijn dan bij mannen. Dat maakt het ook moeilijker. Daarom is het heel erg belangrijk om heel erg zorgvuldig de risicofactoren in kaart te brengen die een andere impact kunnen hebben voor vrouwen en vergeet u daarbij het niet belang van sociale steun. En tot slot, zeg altijd tegen de patiënt als de klachten veranderen, dat ze dan terug moeten komen op uw spreekuur. | What are the main messages I want to send you home with? In the first place: Are you aware that women get cardiovascular complaints as well? Second: That the symptoms of angina pectoris and heart attacks **can** differ from those exhibited by men? This makes it more difficult. That’s why it is important to have clear picture of the risk factors that **may** impact women differently. Don’t forget the role of social support. And finally, always tell the patient to see you again if there’s a change in the complaints. |
| 10:57 – 11:16 | *Mrs. Koning outside in front of practice.* | Mevrouw Koning: Ik ben heel blij dat het weer zo goed met mij gaat. De medicijnen die ik heb gekregen, daar heb ik echt baat bij. En ik vind ook heel fijn dat ik er met mij man over heb gesproken. Wij doen nou ook weer meer dingen samen. En ik kan weer fietsen. | Mrs. Koning: I’m very glad I’m doing well again. I really feel the effects of the medication. I am also very glad I talked to my husband. We do things together more often now. And I can ride my bike again. |
